# Family physician and endocrinologist coordination as the basis for diabetes care in clinical practice

**DOI:** 10.1186/1472-6823-8-9

**Published:** 2008-07-31

**Authors:** Alejandra Duran, Isabelle Runkle, Pilar Matía, Maria P de Miguel, Sofia Garrido, Emilio Cervera, Maria D Fernandez, Pilar Torres, Tomas Lillo, Patricia Martin, Lucio Cabrerizo, Nuria Garcia de la Torre, Jose R Calle, Jose Ibarra, Aniceto L Charro, Alfonso L Calle-Pascual

**Affiliations:** 1 Servicio de Endocrinología y Nutrición, Hospital Clínico San Carlos, Madrid, Spain; 2 Unidad de Formación e Investigación de Atención Primaria, Área 7, Madrid, Spain; 3 Dirección Médica, coordinación atención primaria-especializada, Hospital Clínico San Carlos, Madrid, Spain

## Abstract

**Background:**

To estimate the proportion of diabetic patients (DPts) with peripheral vascular disease treated at a primary health care site after an endocrinologist-based intervention, who meet ATP III and Steno targets of metabolic control, as well as to compare the outcome with the results of the patients treated by endocrinologists.

**Methods:**

A controlled, prospective over 30-months period study was conducted in area 7 of Madrid. One hundred twenty six eligible diabetic patients diagnosed as having peripheral vascular disease between January 2003 and June 2004 were included in the study. After a treatment period of three months by the Diabetes team at St Carlos Hospital, 63 patients were randomly assigned to continue their follow up by diabetes team (Group A) and other 63 to be treated by the family physicians (FP) at primary care level with continuous diabetes team coordination (Group B). 57 DPts from Group A and 59 from Group B, completed the 30 months follow-up period. At baseline both groups were similar in age, weight, time from diagnosis and metabolic control. The main outcomes of this study were the proportion of patients meeting ATP III and Steno goals for HbA1c (%), Cholesterol, HDL cholesterol, LDL cholesterol, triglycerides, blood pressure, albumine-to-creatinine excretion ratio (ACR), body mass index (BMI), waist circumference (WC), anti-aggregation treatment and smoking status.

**Results:**

At the end of the follow up, no differences were found between the groups. More than 37% of diabetic patients assigned to be treated by FP achieved a HbA1c < 6.5%, more than 50% a ACR < 30 mg/g, and more than 80% reached low risk values for cholesterol, LDL cholesterol, triglycerides, diastolic blood pressure and were anti-aggregated, and 12% remained smokers. In contrast, less than 45% achieved a systolic blood pressure < 130 mm Hg, less than 12% had a BMI < 25 Kg.m-2 (versus 23% in group A; p < 0.05) and 49%/30% (men/women) had a waist circumference of low risk.

**Conclusion:**

Improvements in metabolic control among diabetic patients with peripheral vascular disease treated at a primary health care setting is possible, reaching similar results to the patients treated at a specialized level. Despite such an improvement, body weight control remains more than poor in both levels, mainly at primary care level. General practitioner and endocrinologist coordination care may be important to enhance diabetes management in primary care settings.

**Trial registration:**

Clinical Trial number ISRCTN75037597

## Background

Cardiovascular morbidity is a major burden in subjects with type 2 diabetes mellitus. The estimated risk of death is two to six times fold compared with the non-diabetic population. Intensive treatment of multiple modifiable risk factors including hyperglycemia, hypertension, dyslipidemia, obesity, waist perimeter, physical inactivity and smoking reduces micro and macrovascular events [1-3].

Data from the Steno 2 study [3] show that a multifactorial intervention involving multiple risk factors significantly reduces the risk of both cardiovascular and microvascular events. However, other approaches are possible. Patients assigned to conventional treatment were treated by their general physicians (GP). Meanwhile these assigned to the intensive group were followed up by a specialized team at the Steno Diabetes Centre. Glycemic targets during last period of follow-up were similar in both groups. Despite suboptimal glycemic control in both groups, GPs were unable to achieve low-risk values in a similar percentage of patients as the specialized centre, indicating scope for diabetes management at primary care level to be improved. Inadequate glycemic and modifiable risk factors control are due both to patient non adherence, and to the failure of providers to initiate or intensify the therapy appropriately [4-8]. In addition, GP care without well developed support for family doctors was associated with adverse outcomes for diabetic patients. In shared care schemes featuring more intensive support there was no difference in mortality between care in hospital and care in general practice, glycated haemoglobin tended to be lower in primary care and losses to follow up were significantly lower in the primary care setting [9]. Surveys in Spain revealed that metabolic control of diabetic subjects treated at primary care level [10-15] was worse than in diabetes centres [16,17]. These finding suggested that diabetes management in primary care level might be improved if diabetes specialist strategies were implemented by GP.

Since 1993 a foot care programme for people with diabetes and neuropathy is available in Area 7 of Madrid in order to reduce lower extremity amputations [18-20]. Patients with peripheral vascular disease (PVD) are likely to be influenced more favourably by surgical procedures and multifactorial management. We have recently reported that diabetic subjects with PVD have a poorer control of modifiable risk factors than diabetic subjects with other clinical manifestations of cardiovascular disease [21]. Bearing in mind that most of the type 2 diabetes patients are treated in a primary care setting, and that a better metabolic control can reduce their complications, a reduction in the burden of diabetes mellitus should be achieved with a more efficient implementation of multifactorial management at primary level. In addition, general practitioner and endocrinologist coordination should be the basis for diabetes care.

Since 2003 the Foot Care programme in Area 7 of Madrid includes also specific strategies to improve the management of diabetic patients with PVD by empowering family physicians (FP) at primary health centres to carry out multifactorial pharmacologic therapy. Meanwhile a smooth and continuous contact between FP and endocrinology service physicians is maintained.

The impact of these interventions on metabolic control of diabetic patients with PVD treated in a Primary Health Care Level was prospectively assessed over 30 months and the outcomes compared with those patients followed by endocrinologists. In these circumstances we think that FP may achieve follow up and metabolic control at least as good as endocrinologists.

## Methods

The National Health Service-covering 99% of the total population- has divided the Madrid Community into 11 health care areas. The public health care system from the area 7, Madrid, is provided by a single hospital (Hospital Clinico San Carlos) with a single specialized service for in- and out-patients with diabetes mellitus (Department of Endocrinology and Nutrition), and by 22 Primary Health Care Centres with 304 Family Physicians (FP). According to 2004 census, the total urban population in this area was 569,307 (261,529 men and 307,778 women). Since 1991, a Diabetes Programme is available in Area 7. Thus, FP and endocrinologists are coordinated in order to schedule the diabetes care in the out patient setting. Since 2003, a specific multifactorial intervention (MI) programme in type 2 diabetic patients is being carried out. All FP received yearly orientation about the trial in three days -8 hours a day-meeting at central level (CAP Espronceda) with lectures and practical case discussions of MI management. Four meeting were carried out between 2003 and 2006. In addition, three 2-hours sessions at each Health Care Centre were carried out 9 times a year, where FP from three to seven health care teams joined to physicians from the endocrinology service to discuss Steno-based algorithms developed by endocrinology and FP leaders of each Health Care Centre. In total, 216 hours sessions were carried out between 2003 and 2006. Theses algorithms were evaluated for clinical acceptability, considering items such as patient situation, self-monitoring of capillary blood glucose, laboratory and clinical data. FP also had an open and continuous communication with endocrinologists by mail, phone and face to face. In addition, each FP has a personal endocrinologist assigned for contact. The main focus of this program is not a single contact, but a continuous exchange of information between FP and the diabetes team, to optimize the management of these diabetic patients and avoid inertia.

The recommendations for management of hyperglycaemia, hypertension and dyslipidemia were based upon the Steno-2 study and body weight and waist circumference after ATP-III targets. In addition a stepwise implementation of pharmacological treatment and behaviour modification to achieve low risk cardiovascular values and to smoke cessation if necessary was applied. In short, patients were periodically followed, at least every 2 to 4 weeks in the beginning and after each pharmacologic treatment change, in order to adapt their therapy to the following objectives: -blood pressure < 130/80 mm Hg, -LDL cholesterol < 100 mg/dl, -triglycerides < 150 mg/dl, -HDL cholesterol > 40/50 mg/dl (M/W), -fasting and pre-prandial capillary glucose values between 70 and 120 mg/dl -and 2 h-postprandial capillary glucose values between 70 and 135 mg/dl in 60% of the capillary blood glucose determinations, as well as Hb A1c (DCCT standardized) < 6.5%. Simultaneously, they were antiaggregated (100 mg aspirin) and a smoking-cessation program was offered. Nutrition intervention based on Diabetes Nutrition and Clinical Trial study, DNCT [16,17,22] aimed to achieve PUFAs/SFAs > 0.4 and MUFAs/SFAs > 1.4. Regular leisure light-exercise was recommended, namely climbing at least 4 flights of stairs and walking at least 4 blocks 4 times a day each. In addition, irbesartan 300 mg/day was prescribed when the albumin-to-creatinine ratio > 30 mg/g, irrespectively of the blood pressure level. After this period, an individual medical consultation every third month (4 a year) was offered to all patients. Nurse (and medical if necessary) consultations were always open at patients' request.

The protocol of type 2 diabetes management and diabetes and cardiovascular disease treatment was approved by the Commission of Clinical Guidelines of the St Carlos University Hospital. All patients received treatment according to these guidelines which are annually revised. An informed consent was considered not necessary. The study was conducted according with the Declaration of Helsinki and with the consort guidelines for clinical trial. The study was approved by the St Carlos University Hospital Ethic Committee.

Between January 2003 and June 2004, 924 diabetic patients recruited for the screening foot-care programme were tested for peripheral vascular disease and selected when diagnosed. Peripheral vascular disease was considered when diabetic subjects had at least one diagnostic criteria: -patients who underwent a peripheral vascular revascularization at least 6 months before, -patients with previous non-neuropathy foot lesions at least 6 months before, or -at least one ABI < 0.8. The design of the screening programme focused on detection of early neuropathy and the intervention programme based on continuous well-organized education, and supported by regular podiatry assistance for people with diabetes with different stages of neuropathy, has been previously described elsewhere [19,20]. For sample size calculation for the hypothesis that there will be no difference between FP and specialist care, a primary composite end-point difference of percentage of diabetic patients with progression of peripheral vascular disease and mortality during planned median follow-up period of 60. months has been used. Sample size adjusted for drop-outs of 120 patients powered to detect a 20% difference between both groups. For this planned middle-road 30-months analysis, the sample size was estimate in two ways. For the hypothesis that there will be no difference between FP and specialist care in Steno and ATP III goals, a primary end-point difference in HbA1c value has been used in order to estimate sample size. From previous studies (UKPDS, DCCT, Steno) differences expected in HbA1c levels between control group and experimental group were about 0.7%, being 10% of HbA1c baseline values in our study. With 41 subjects in each group, the study had 90% power at 5% significance (2-sided) for difference = 0 versus difference = 0.72, to detect a clinically significance difference (10%) in the HbA1c value between both FP group and specialist care group. In addition we estimated sample size for the hypothesis that there will be no inferior FP treatment in relation to specialist care. A primary end-point difference of percentage of diabetic patients achieving HbA1c < 6.5% more than 20% has been used. In this case, with 60 patients in each group, the study had 89% power at 5% significance to detect a clinically significance difference (20%) to be no inferior FP group versus specialist care group.

**Figure 1:**
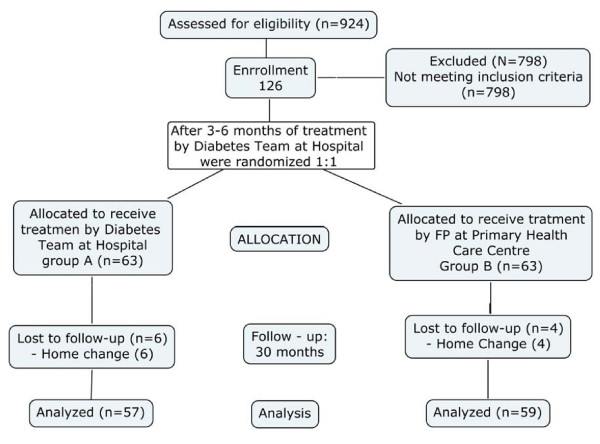
Structure of the clinical trial.

126 eligible diabetic patients were diagnosed as having peripheral vascular disease. After a treatment period of 3–6 months in the Diabetes Unit of the St Carlos Hospital, 63 patients were randomly (1:1) assigned to receive treatment from family physicians at primary care level (4 were lost during the follow-up due to address home change) and 63 from the diabetes team at the Endocrinology Service of the St Carlos Hospital (6 were lost during the follow-up due to address home change). One hundred sixteen patients, 57 treated by the diabetes team at hospital (Group A), and 59 treated by FP at primary Health Care Centre (Group B), completed the 30 months of follow-up and were analyzed. The structure of the clinical trial is shown in figure 1, and clinical data of analyzed patients are displayed in table 1.

**Table 1:** Characteristic of the survey population by group

	Group A	Group B
N (Men/Women)	57 (43/14)	59 (39/20)
Age (yr.)	69 (58–74)	70 (57–76)
Duration of disease (yr)	19 (10–26)	19 (10–28)
Diabetes complications n (%)		
Diabetic retinopathy	35 (61)	33 (56)
Diabetic neuropathy	44 (77)	47 (80)
Diabetic nephropathy	26 (46)	33 (56)
Diabetes Treatment n (%)		
Oral agents	18 (32)	20 (34)
Insulin	41 (72)	37 (63)
Lipid lowering Treatment n (%)		
Statins	44 (77)	35 (59)
Fibrates	2 (4)	7 (12)
Antihypertensive treatment n (%)		
ACE inhibitors or ARA II	51 (88)	46 (78)
3 or more drugs	7 (12)	11 (19)
Antiaggregation n (%)	50 (88)	53 (90)
Actual smokers n (%)	11 (19)	11 (19)
Inclusion criteria n (%)		
Arterial reconstruction	27 (47)	30 (51)
Foot ulcer	16 (28)	12 (20)
ABI < 0.8	14 (25)	17 (29)

Group A, diabetic patients treated by endocrinology service. Group B, diabetic patients treated by coordinated team, Family Physicians (FP) and endocrinologist, at primary care site.

Data expressed as median (Q1–Q3) or n (%).

ABI, ankle-brachial index. ACE, angiotensin convert enzyme ARA II, angiotensin II-receptor antagonist.

Visits at the beginning of the study and every 6 months until completing a 30 months follow-up period included a physical exam and laboratory tests. Two nurses were responsible for these visits. Body weight (barefoot, with indoor clothes), waist circumference and blood pressure with adequate sized armlet after 3 minutes in a supine position were measured. At the same time three consecutive first-morning urine samples were collected for the analysis of albumine-to-creatinine ratio (ACR). Blood samples after at least 10 h fasting were obtained to determine HbA1c (DCCT standardized), total cholesterol, HDL cholesterol, triglycerides, Apolipoprotein A1, Apolipoprotein B, and lipoprotein (a) levels.

The cost associated to the continuous education programme was determined by taking into account the number of hours of sessions. According to Lain Entralgo Agency the cost of 1-hour session was 72.5 € during 2003 and 2004 and 85 € during 2005 and 2006. In total 312 hours sessions were carried out during the study.

The statistical study was performed by using the SPSS 12.0 program for windows. Descriptive data are expressed as median and quartiles 25 and 75 for describing the studied variables. Parametric, one-way analysis of variance and non-parametric Mann-Whitney and Kruskall-Wallis tests to determine whether there are significant differences between two or more independent groups were carried out.

## Results

The median of clinical and laboratory data change at entry and during follow-up were similar among diabetic patients assigned to be treated by the diabetes team (Group A) or by family physicians (Group B) and differences between groups were not found (table 2). A significant trend to reduce the medians of the blood pressure values (SBP, -10 mm Hg in Group A and B; DBP – 4 mm Hg in Group A and 7 mm Hg in Group B, respectively), total cholesterol (Group A, 181 vs. 148 mg/dl and Group B, 189 vs160 mg/dl, respectively) and LDL cholesterol levels from 104 to 78 mg/dl (Group A) and from 107 to 81 mg/dl (Group B) were observed in both groups (all p < 0.05). No significant changes in clinical data including BMI, waist circumference, HbA1c, and albumine-to-creatinine ratio values and triglycerides and HDL cholesterol levels were found. The percentage of diabetic patients achieving the blood pressure (SBP: from 21 and 24% to 51 and 43% and DBP: from 53 and 46% to 82 and 86% for Group A and B, respectively), total cholesterol (Group A, 68 vs. 93%; Group B 68 vs. 95%) and LDL cholesterol (Group A, 46 vs. 84%; Group B, 39 vs. 78%) targets increases during the follow-up, whereas glycemic control and triglycerides and HDL-cholesterol levels were similarly maintained in both groups. In addition, BMI (slightly greater in group B as compared with group A, p < 0.05) and waist circumference remain unchanged in both groups and the number of actual smokers and anti-aggregated subjects were similar in both groups. When these figures were compared between group A and B, differences were only found in BMI change (p < 0.05). Data are displayed in table 3.

**Table 2:** Clinical and Laboratory data for the diabetic subjects during treatment period by Group

	Baseline	6	12	18	24	30 months
BMI (Kg.m-2)						
- Group A	28 (25–31)	28 (25–32)	28 (25–32)	28 (25–32)	28 (26–32)	28 (25–32)
- Group B	29 (27–34)	30 (27–32)^&^	30 (27–33)^&^	30 (27–32)^&^	30 (28–36)^&^	30 (27–35)^&^
Waist Circumference(cm)						
- Group A						
Men	103 (96–109)	104 (98–109)	102 (97–108)	102 (92–109)	103 (97–109)	103 (97–112)
Women	100 (88–114)	96 (87–113)	95 (86–110)	98 (81–113)	99 (81–114)	98 (80–115)
-Group B						
Men	103 (99–113)	106 (98–115)	106 (98–113)^&^	103 (99–114)	103 (98–111)	104 (95–118)
Women	100 (89–114)	104 (84–118)^&^	110 (83–128)^&^	107 (82–125)^&^	101 (78–127)	99 (79–126)
SBP mm Hg						
- Group A	140 (135–155)	137 (130–144)	135 (125–144)*	135 (125–145)*	135 (125–144)*	130 (125–135)**
- Group B	145 (132–160)	138 (128–150)*	135 (127–150)*	132 (125–144)**	135 (125–145)**	135 (121–147)*
DBP mm Hg						
- Group A	80 (75–87)	76 (70–80)*	75 (70–80)*	78 (70–80)	78 (70–80)	76 (71–80)*
- Group B	85 (76–90)	79 (70–84) *	78 (71–80)*	77 (70–79)*	77 (69–78)*	78 (68–79)*
HbA1c (%)						
- Group A	7.2 (6.5–8.5)	7.0 (6.6–7.9)	7.0 (6.6–7.8)	6.9 (6.4–7.8)	7.1 (6.4–7.9)	7.3 (6.5–7.9)
- Group B	7.5 (6.5–9.2)	7.3 (6.3–8.2)	7.4 (6.4–8.0)	7.0 (6.3–8.2)*	7.3 (6.4–8.0)	7.1(6.4–8.3)*
ACR (mg/g)						
- Group A	22 (8–145)	20 (8–99)	22 (9–66)	15 (9–76)	19 (8–86)	27 (9–97)
- Group B	25 (7–128)	29 (10–137)	25 (8–135)	25 (8–141)	20 (11–105)	25 (9–158)
Total Cholesterol (mg/dl)						
- Group A	181 (158–208)	165 (151–182)*	165 (150–183)*	155 (135–175)*	154 (129–166)*	148 (135–163)**
- Group B	189 (164–217)	167 (151–200)*	170 (147–185)*	159 (141–178)**	158 (132–182)**	160 (143–185) **
HDL-Cholesterol (mg/dl)						
-Group A						
Men	46 (41–55)	46 (43–56)	50 (43–57)	50 (43–56)	46 (40–54)	48 (40–55)
Women	55 (48–65)	59 (51–71)	58 (49–65)	64 (50–66)	63 (56–68)	56 (54–64)
-Group B						
Men	49 (39–54)	49 (42–55)	50 (44–53)	50 (43–54)	50 (47–55)	50 (45–55)
Women	50 (42–60)	53 (45–64)	45 (43–58)	48 (42–58)	48 (37–55)	52 (43–57)
LDL-Cholesterol (mg/dl)						
- Group A	104 (87–128)	93 (83–107)	88 (75–104)*	77 (66–95)**	75 (64–94)**	78 (69–86)**
- Group B	107 (87–136)	96 (78–112)*	91 (77–109)*	82 (72–99)**	80 (64–98)**	81 (70–97) **
Triglycerides (mg/dl)						
- Group A	102 (85–145)	98 (74–149)	112 (77–139)	108 (71–138)	100 (80–141)	97 (75–137)
- Group B	119 (86–160)	105 (87–160)	109 (80–160)	116 (79–158)	117 (79–152)	105 (79–150)

Group A, diabetic patients treated by endocrinology service. Group B, diabetic patients treated by coordinated team, FP and endocrinologist, at primary care site. Data expressed as median (Q1–Q3).

BMI, body mass index. SBP, Systolic Blood Pressure. DBP, Diastolic Blood Pressure. ACR, albumine-to-creatinine ratio.

*p < 0.05, ** p < 0.01, denote differences in relation to baseline values. ^&^p < 0.05 denote differences between Group A vs. Group B.

**Table 3:** Number (%) of DPts on Targets of Metabolic Control according to ATP-III Panel and Steno Study

	Baseline	6	12	18	24	30 months
HbA1c < 7%:						
- Group A	25 (44)	31 (55)	32 (56)	34 (60)	28 (49)	24 (42)
- Group B	19 (33)	22 (37)	27 (46)	31 (53)	25 (43)	27 (46)
HbA1c < 6.5%:						
- Group A	16 (28)	14 (25)	13 (23)	21 (37)	17 (30)	21 (37)
- Group B	15 (26)	16 (27)	18 (30)	24 (41)	22 (37)	22 (37)
SBP < 130 mm Hg:						
- Group A	12 (21)	21 (37)	24 (42)*	22 (39)*	23 (41)*	29 (51)*
- Group B	14 (24)	17 (29)	28 (48)*	28 (48)*	24 (41)*	25 (43)*
DBP < 80 mm Hg:						
- Group A	30 (53)	44 (77)	49 (86)*	44 (77)	45 (79)*	47 (82)*
- Group B	27 (46)	44 (75)	50 (85)*	53 (90)*	53 (90)*	51 (86)*
Cholesterol < 200 mg/dl:						
- Group A	39 (68)	50 (88)*	51 (90)*	48 (84)*	54 (95)*	53 (93)*
- Group B	40 (68)	45 (76)*	48 (81)*	52 (88)*	55 (93)*	56 (95)*
Triglycerides < 150 mg/dl:						
- Group A	44 (77)	44 (77)	48 (84)	48 (84)	47 (82)	50 (88)
- Group B	45 (76)	40 (68)	42 (71)	43 (73)	45 (76)	48 (81)
HDL cholesterol (mg/dl)						
Men > 40:						
- Group A	33 (77)	33 (77)	37 (86)	39 (91)	32 (74)	30 (70)
- Group B	28 (72)	31 (80)	31 (80)	31 (80)	30 (77)	34 (87)
Women > 50:						
- Group A	9 (64)	11 (79)	10 (71)	10 (71)	12 (86)	12 (86)
- Group B	10 (50)	12 (60)	9 (45)	8 (40)	8 (40)	11 (55)
LDL cholesterol < 100 mg/dl:						
- Group A	26 (46)	41 (72)*	38 (67)*	44 (77)*	50 (88)**	48 (84)**
- Group B	23 (39)	33 (56)*	37 (63)*	46 (78)*	51 (86)**	46 (78)**
Apolipoprotein B < 100 mg/dl:						
- Group A	32 (56)	45 (79)*	48 (84)*	48 (84)*	52 (91)*	53 (93)*
- Group B	27 (46)	42 (71)*	42 (71)*	51 (86)*	50 (85)*	50 (85)*
BMI < 25 Kg.m-2:						
- Group A	13 (23)	12 (21)	11 (19)	13 (23)	12 (21)	13 (23)
- Group B	5 (9)^&^	5 (9)^&^	5 (9)^&^	9 (15)^&^	7 (12)^&^	7 (12)^&^
Waist Circumference						
Men < 102 cm:						
- Group A	21 (49)	18 (42)	22 (51)	23 (53)	20 (47)	20 (47)
- Group B	17 (44)	18 (46)	16 (41)^&^	19 (49)	19 (49)	19 (49)
Women < 88 cm:						
- Group A	3 (21)	4 (29)	5 (36)	4 (29)	4 (29)	4 (29)
- Group B	4 (20)	5 (25)	5 (25)	6 (30)	6 (30)	6 (30)
ACR < 30 mg/g:						
- Group A	31 (54)	32 (56)	32 (56)	34 (60)	29 (51)	29 (51)
- Group B	26 (44)	30 (51)	27 (46)	31 (53)	25 (42)	30 (51)
Current smokers:						
- Group A	11 (19)	8 (14)	8 (14)	7 (12)	7 (12)	7 (12)
- Group B	11 (19)	8 (14)	7 (12)	7 (12)	7 (12)	7 (12)
Antiaggregated treatment:						
- Group A	50 (88)	50 (88)	50 (88)	50 (88)	50 (88)	50 (88)
- Group B	53 (90)	53 (90)	53 (90)	53 (90)	53 (90)	53 (90)

Group A, diabetic patients treated by endocrinology service. Group B, diabetic patients treated by coordinated team, Family Physician and endocrinologist, at primary care site.

SBP, systolic blood pressure. DBP, diastolic blood pressure. BMI, body mass index. ACR, albumine-to-creatinine ratio.

*p < 0.05, ** p < 0.01, denote differences in relation to baseline values. ^&^p < 0.05, denote differences between Group A vs. Group B.

In total 4 three-days meetings, one a year (96 hours in total) and 36 three-days 2-hours sessions (216 hours in total), nine a year, were carried out between 2003 and 2006. The excess of cost associated with the programme was estimated in 25,570 €, 6,392.5 € a year.

## Discussion

Cardiovascular events remain the first cause of death in people with diabetes, and multifactorial treatment is the cornerstone to improve outcomes. Treatment of type 2 diabetic patients is mainly provided by the FP. Data in this study suggest that the impact on metabolic outcomes of continuous coordination between FP and the specialized diabetes team allows diabetic patients treated in the primary care setting to obtain similar levels of ATP III treatment and Steno goals than diabetic patients treated by a specialized diabetes team. More than 75% diabetic patients with peripheral vascular disease treated at primary health care level achieved adequate targets for diastolic blood pressure, cholesterol, LDL-cholesterol and triglycerides levels (also apolipoprotein B values). Also more than 35% of patients reached Hb1c level < 6.5%, and most of the patients were anti-aggregated. These figures were maintained at the end of follow up. In addition, there were no differences between these outcomes and those of the patients treated exclusively in the endocrinology service. These percentages of patients in low-risk values are similar to that reported for patients treated by the Diabetes team in the Steno study. However in our study, twice the percentage of patients achieved an HbA1c < 6.5% compared with the Steno group, similar that reported in specialized centres in Spain [16,17], and significantly greater than the reported in primary health settings in Spain, including diabetic patients at low-risk [10-15] and high-risk [21]. Similarly, the achievement of the ADA recommendations among US adults in clinical practice is no better than in the current study [23].

After the Steno 2 results [3], is well recognized that the management of patients in high-risk must simultaneously involve glycemic control as well as other cardiovascular risk factors. However FP may adopt extremely heterogeneous management strategies and many DPts may remain undertreated. In addition, the situation of FP in Spain is usually unstable and frequently change their work place, being an additional barrier to effective care. Along the duration of the present study 234 FP (77%) changed their work place. Usually, diabetic patients followed by different FP in the same health care centre show a risk of inadequate metabolic control similar to that of diabetic patients followed by FP adopting a non-aggressive policy. However, our data do not support that, showing that a primary-secondary interacted care as designed in our area may result in better clinical outcomes in terms of metabolic control. Considering that the vast majority of diabetic patients should be treated and followed at the primary health care level, several strategies were designed [24]. In order to improve patients outcomes, physicians-centered educational activities to increase the awareness of the potential benefits of a tight multifactorial control were applied in our study. At the beginning, patients were treated by endocrinologists as usually during a 3–6 months period, in order to adapt the pharmacological treatment and behaviour modification at least every 2 to 4 weeks, and then by family physicians. However, family physicians and endocrinologists were continuously in contact. Guidelines for multifactorial intervention were the same for both FP and endocrinologists. Continuous formative evaluation of diabetes teaching and treatment goals has been reported as essential [25], as considered in our programme. Training and coordination of clinical programmes with FP are included in the specialist consultant's job description. According to data reported in this study, the levels of most of the therapeutic targets remained stable or improved during the follow-up, in a similar way in both groups of patients. In order to improve the programme and to identify possible causes of failure and inertia, several strategies were developed. A continuous exchange of information between FP and endocrinologists was established across face to face meetings, mail or phone contact. It is quite likely that the results obtained in the present study have been favourably influenced by this coordination between medical and educators teams.

The aim of treatment of overweight type 2 diabetic patients must be reduction of body weight as well as other cardiovascular risk factors. Despite the improvement in cardiovascular risk factors, control of body weight remains inadequate as previously reported by our group [26]. Possibly, both FP and specialized physicians give greater priority to the control of other metabolic factors because these patients present greater difficulty in losing weight compared to diabetic subjects without peripheral vascular disease, thus implying the need for pharmacological treatment. In Spain, the National Health System covers between 75% and 100% of the cost of the pharmacological treatments when prescribed by physicians. Sibutramine and orlistat are excluded and have to be paid by the patients. In our study none received anti obesity drugs. In addition, educational programmes using behaviour modification are useful to induce weight reduction, as our group previously reported [26], but physical exercise remains as the main long-term predictor for weight loss [27], and DPts with PVD have a reduced exercise capacity. Surgical procedures in Spain is not considered when BMI < 40 Kg.m-2, as our patients.

The present study findings are potentially important because the management of type 2 diabetic patients in Spain is mainly provided by the FP. When targets of diabetes treatments are not reached, diabetic patients should be derived to the specialized level. Nevertheless, this measure is usually taken too late. To avoid this failure, in Area 7 of Madrid a system of immediate communication through phone, mail or face to face is available. In addition, periodic meetings with health care teams and their leaders have been established. The diabetes programme in Area 7 of Madrid defines how diabetes management should be more that who is responsible for it. In our pattern, care of diabetes mellitus should be shared between FP and the diabetes team. The success of this strategy relays in guaranteeing a continuous communication between both FP and the diabetes team. FP had full access to nurse specialists in foot programme and diabetes education, dietitians and podiatry depending on the foot unit. The decrease in the clinical burden in the specialized level translates in more availability for clinical research and for assistance of clinical complex cases. Training and coordination of clinical programmes with FP are included in the endocrinologist consultant's job description, without to increase the resources. As to the economic cost, the two hours-sessions and the three days annual meetings, were taken into account to estimate the extraordinary expenses. Therefore, according to data obtained in the current study, we may consider that this programme of continuous education is efficient.

## Conclusion

In summary, our results show that a training and continuous communication between primary care and endocrinology is possible in clinical practice and results in benefits in terms of health care and cardiovascular risk factors control, with an appropriate use of personal resources. A reduction in diabetes complications as well as cardiovascular events will be expected.

## Availability

Clinical Trial number ISRCTN75037597 available at 

## Abbreviations

ACR: albumin-to-creatinine excretion ratio; BMI: body mass index; WC: waist circumference; PVD: peripheral vascular disease; FP: family physicians; MI: multifactorial intervention; DNCT: Diabetes Nutrition and Clinical Trial study; ABI: ankle-brachial index; ACE: angiotensin convert enzyme; ARA II: angiotensin II-receptor antagonist; SDP: systolic blood pressure; DSP: diastolic blood pressure.

## Competing interests

The authors declare that they have no competing interests. The foot care programme was partially supported by grants from the European Union, Sociedad Española de Endocrinología y Nutrición, Fundación Fernandez Cruz and Fundación del Servicio de Endocrinologia y Nutrición.

## Authors' contributions

The paper was written on behalf of all authors by AD, JRC, NGT and ALC-P. All authors provided detailed comments on the paper at all stages of drafting and assisted with the interpretations of the results. PM, MPdeM, SG, EC, JRC and ALC-P played the major role in coordinating the study. IR, PM and ALC-P participated on continuous communication by mail. All authors participated on continuous contact by phone or face to face. ALC-P, MPdeM, PM, JRC, LC, IR and MDF participated in the two-hours sessions at each Health Care Centre, and three days meeting. AD, JI and ALC-P participated in the screening foot programme. ALC-P, IR, PT, TL and Ach participated in the design and coordination.

## Pre-publication history

The pre-publication history for this paper can be accessed here:


